# Enfuvirtide biosynthesis in thermostable chaperone-based fusion

**DOI:** 10.1016/j.btre.2022.e00734

**Published:** 2022-05-14

**Authors:** Vladimir Zenin, Maria Yurkova, Andrey Tsedilin, Alexey Fedorov

**Affiliations:** Bach Institute of Biochemistry, Research Center of Biotechnology of the Russian Academy of Sciences, Russia

**Keywords:** Molecular chaperones, Fusion system, Peptide biosynthesis, Protein engineering, Thermostable protein

## Abstract

Synthetic peptides are in high demand as biologically active substances. Solid phase synthesis is the primary method of peptide production. However, it has drawbacks: large amount of chemical waste and rapid increase in price with peptide length. Biosynthesis is intended as method to bypass these flaws. Direct biosynthesis is usually not effective and among other approaches for improving quality and quantity of target product fusion partners are widely used. In this study we used a thermostable chaperon-based fusion partner developed by us to produce enfuvirtide in *Escherichia coli* expression system. Fusion partner's thermal stability provided additional purification mode by thermal denaturation of host proteins in lysate. Fusion protein was purified by ion exchange chromatography after lysate heating step and was then hydrolyzed with cyanogen bromide to release enfuvirtide. Enfuvirtide was isolated by RP-HPLC up to 94% purity with total yield of 2.86–3.31 mg per 1 L of low-density culture. The data demonstrate the posibility of thermostable chaperone-based fusion partner GroEL use for effective peptide biosynthesis.

## Highlights

• Peptide biosynthesis in thermostable chaperone-based fusion
• Model peptide enfuvirtide sequence inserted between Ser 199 and Tyr 201 of modified GroEL chaperone
• Host proteins thermal denaturation as purification step
• Methionineless fusion partner and cyanogen bromide treatment to produce free peptide

## 1 Introduction

Peptides have great potential as biologically active substances: their secondary structure allows them to act on complex membrane receptors; natural peptide hormones and neurotransmitters are "blueprints" for new peptide pharmaceuticals, peptide degradation products are included in the human body's metabolism. Also, besides high clearance rate, they penetrate deeper in tissues and can be synthesized much cheaper than conventional antibodies [1].

Modern chemical synthesis methods allow to make polypeptides more than 100 amino acid residues long. For example, Genescript offers the synthesis of polypeptides up to 200 amino acid residues long as a routine service. However, the cost of the resulting drug usually limits its use. To date, the introduction of peptides with a size of more than 40 amino acid residues into clinical practice is obviously limited [2].

Usually, large peptides are synthesized by the combination of SPPS and ligation methods. The SPPS efficiently produces up to 40 aa-long peptides and native ligation helps to unite such fragments into an even longer peptide chain. Both methods have their own weak spots.

There are some hydrophobic sequences with SPPS synthesis difficulties [3]. Sequences that cannot be synthesized by the SPPS method have been described: amylin (1–37) [4,5]; amyloid beta peptide (1–42) [6,7] and influenza B M2 proton channel (1–51) [8].

Native ligation and extended methods use wide variety of concepts and mechanisms, each with its own features. Usually, the process is slow and sensitive to amino acids on merging ends [9].

HPLC purification of synthesized peptides is difficult and pricey. There are some ways to make it cheaper. One of them, and straightforward, is to make the synthesis process less prone to produce highly similar products. Biosynthesis is much more accurate [10,11] than SPPS. Respectively, for 40 aa biosynthetic peptide average single-substituted impurities content is about 4% or less. Enfuvirtide, the best industrial example of SPPS, gives ∼75% HPLC purity of crude peptide [12]. Typical and most abundant peptide impurities are closely related [13]. Usually, related impurities are sequences with deletion, truncation and incompletely unprotected sequences, miscleavage and side-reaction products.

An alternative for SPPS is biosynthesis. Peptides are usually difficult to synthesize standing alone in their mature form in a bacterial expression system because of their activity toward host cell or degradation by cellular proteases. The most efficient strategies for peptide biosynthesis are expression in tandem repeats or in fusion with a well-expressed partner, or both. We should note ketosteroid isomerase as the only commercially available fusion system for inclusion bodies formation and because of it's similar to our fusion partner advantages for target peptide. The ketosteroid isomerase forms inclusion bodies, thus facilitating purification, and giving the protection of the cell from peptide toxicity and of the peptide from proteolysis [14].

Earlier, we developed a fusion partner [15,16] for the biosynthesis of a toxic antibacterial peptide polyphemusin I in *E. coli* as part of a fusion protein. Target peptide was inserted into thermostable chaperone GroEL sequence and co-expressed with GroES co-chaperone. The insert was placed in such a way that the peptide protruded inside the substrate binding cavity of the assembled GroELS particle. The main features of this fusion were the expression of the fusion protein in stable soluble form, peptide protection from the bacterial internal environment, protection of bacteria itself from synthesized peptide's toxicity and the possibility of purification from host proteins by lysate heating.

It should be mentioned that the proposed fusion partner is intended only for biosynthesis of peptides consisting of 20 encoded amino acids. Cyanogen bromide hydrolysis, if used, additionally restricts the use of methionine in peptide sequence. The methionine link between fusion partner and target peptide can be modified to facilitate other cleavage protocols. The expanded genetic code and chemical or enzymatic modification of synthesized peptide probably are workaround to produce some NRPs and RiPPs but it is out of scope of the current work.

The main goal of this study is to show the applicability of the same fusion partner for a practically valuable peptide with differing properties, enfuvirtide. In contrast to polyphemusin I, enfuvirtide is a large, hydrophobic and low pI peptide. It is an active pharmaceutical ingredient, used against HIV I virus, and is chemically synthesized on a large scale. Despite its state-of-the-art synthesis, one dose at the time of this study costed more than $70 with the necessity of twice a day administration.

Additionally, we refined the purification and hydrolysis protocol [16] aiming to make it easy and scalable for possible practical use.

## 2 Materials and methods

### 2.1 Chemicals

LB tissue culture grade, buffer components and SDS-PAGE reagents “for biochemistry” grade, by Amresco, USA; Pierce Unstained Protein MW Marker by Thermo Fisher Scientific, USA; formic acid for biochemistry by AppliChem, USA and MS-grade solvents by Merck, Germany were used.

### 2.2 Enfuvirtide gene synthesis and cloning

Enfuvirtide gene (ATGTATACCAGCCTGATTCATAGCCTGATTGAAGAAAGCCAGAACCAGCAGGAAAAAAACGAACAGGAACTGCTGGAACTGGATAAATGGGCGAGCCTGTGGAACTGGTTTATG) with BamHI at 5’-end and EcoRI at the 3’-end restriction sites were synthesized by GeneCust, Luxembourg.

The previously constructed bicistronic vector ploop/ES was made on pETDuet-1 plasmid base [15]. Briefly, on MCS-1 was cloned *T. thermophilus* co-chaperone GroES gene (Accession: AAS82055.1) and on MCS-2 was cloned a modified *T. thermophilus* chaperone GroEL gene (Accession: CAB65482.1) with all methionines replaced with leucines and a new polylinker BamHI, HindIII and EcoRI introduced between triplets encoding Ser 199 and Tyr 201.

The plasmid ploop/ES and the synthetic gene were digested with BamHI and EcoRI (Thermo Scientific, USA) and ligated with T4 DNA ligase (Thermo Scientific, USA). All operations were performed in provided buffers according to enzymes’ user manuals.

### 2.3 Transformation and strain storage

5 mL LB was inoculated with overnight *E. coli* culture ad incubated at 37°С till OD600 0.5±0.1. Culture was cooled to 4°С centrifuged at 3000 g 4 min 5418R (Eppendorf, Germany). Cells were washed twice with ice-cold water by resuspension and centrifugation then suspended in 50 µL ice-cold water 0.5 µL of plasmid DNA (10–15 ng) was added and pulsed with MicroPulser Electroporator (Bio-Rad, USA) at 2.5 kV in cooled 2mm cuvette. 1 mL LB was inoculated with transformed cells and incubated for 1 h, then transferred to LB agarose plate with ampicillin (100 μg/mL).

Overnight culture was collected from the agar surface and resuspended in 1:1 80% (v/v) glycerol – LB and stored at -80 °С, MDF-U76V (Panasonic, Japan).

Stored culture was sampled at -20 °С rack in laminar air flow BMB-Laminar C (Lamsystems, Russia).

### 2.4 Cultivation, expression and lysis

LB medium containing ampicillin (100 μg/mL) was inoculated with stored cells and incubated at 37 °С overnight. 2.5 L of LB containing 100 μg/mL ampicillin was inoculated with 30 mL night culture and incubated till OD600 0.5±0.1 IPTG was added in 1M water stock to a final concentration 0.4 mM and incubation continued for 3 h. Cell culture was cooled to 4°С and harvested by centrifugation at 3000g 15 min CR22N (Hitachi, Japan) then washed with ice-cold 50 mM Tris buffer pH 7.5 containing 150 mM sodium chloride (TBS) by resuspension and centrifugation. Cells were stored at -20 °С.

Stored cells (about 3.6 g) were defrosted at 4°C, resuspended in 300 mL TBS with the addition of 5 mM EDTA and 1 mM β-mercaptoethanol and sonicated in ice bath in high glass beaker by Q500 (Qsonica, USA) with 100% amplitude, 10 s with 30 s intervals and total 300 J/mL applied.

Lysed cells were centrifuged at 4°С, 16,000g 35 min CR22N (Hitachi, Japan), supernatant was collected.

The supernatant was loaded in glass beaker and with constant vigorous stirring, rapidly (15 min) heated to 65 °С from 4 °С, treated 5 min at 65 °С and cooled (20 min) to 4 °С. The heated sample was centrifuged again in the same conditions. The supernatant was collected and pellets discarded.

### 2.5 Ion exchange and buffer exchange

NGC Discovery 10 (Bio-Rad, USA) chromatography system was used, 215, 255, 280 nm wavelengths and conductivity were recorded.

Pre-treated lysate was diluted with 3 volumes of 5 mM EDTA and 1 mM β-mercaptoethanol and filtered through Porafil RC-0.45, 47 mm, 0.45 µm (Macherey-Nagel, Germany) membrane.

400 mL portions of filtrate were applied on XK 16/20 (GE Healthcare, USA) glass column with pre-eqilibrated 14 mL of WorkBeads 40 DEAE (Bio-Works, Sweden) resin. Column was flushed with 3 CV of 10mM Tris buffer pH 7.5 with 40mM NaCl, 5 mM EDTA and 1 mM β-mercaptoethanol and eluted with 40–500 mM sodium chloride gradient in 10 CV. Fractions were collected and analyzed by SDS-PAGE. GroEL-enfuvirtide-rich fractions were united and buffer was changed to 10mM ammonia acetate pH 7.5 on HiPrep 26/10 Desalting (Cytivia, USA) column according to column manual.

### 2.6 SDS-PAGE and densitometry

Standard 10% or 10–20% gradient protocol was used with Coomassie Brilliant Blue R-250 staining [17].

Gels were scanned with Perfection 1600 photo scanner (Epson) at 600 dpi resolution and 8 bit gray scale TIFF were processed with ImageLab 6.0.1 software (Bio-Rad, USA). 0.5–2 µg BSA calibration curve with 95% CI was plotted, based on 2 independently weighted samples. GroEL-enfuvirtide samples were applied intermediately with BSA in the same concentration (w/v). GroEL-enfuvirtide/BSA peak area ratio was decided to be the best approximation of GroEL-enf/BSA primary component content ratio (n=8). Arithmetic means of proteins purity were used to calculate total GroEL- enfuvirtide mass in the sample. The calibration was normalized to protein molecular weight ladder and used for approximate concentration estimation on other gels.

### 2.7 Lyophilization and cyanogen bromide treatment

United fractions in ammonia acetate buffer were frozen on the walls of glass vacuum flask in liquid nitrogen and lyophilized Alpha 3, 4 LSCbasic (Martin Christ, Germany) at 0.05 mBar pressure and -110°C condenser temperature.

Dry protein was collected, weighted with 0.01 mg precision, EX225D (Ohaus, USA), and stored at -20 °С.

Stored protein was warmed up to room temperature and 2-3 mg were weighted with 0.01 mg precision. The protein sample was dissolved 5 mg/mL in 70% (v/v) formic acid. 100 µL of cyanogen bromide 5M stock solution in acetonitrile was added to 1 mL of protein solution. HPLC column thermostat CT 2.1 (Knauer, Germany) was used for temperature control.

The reaction was quenched after one hour by 10-fold water dilution, followed by immediate liquid nitrogen flash-freezing and lyophilization at 0.05 mbar pressure and -110°C condenser temperature.

### 2.8 RP-HPLC purification and hydrolysis and HPLC step yield assessment

Lyophilized reaction products were dissolved in 60% mobile phase in concentration 10 mg of dry mass per ml. The mixture was centrifuged at 4°С, 16,000g 15 min, supernatant was collected and diluted with mobile phase A to 30% mobile phase concentration.

Purification was performed on Azura HPLC system (P6.1L, DAD 2.1L, AS 6.1L, CT 2.1) (Knauer, Germany) with Foxy R1 fraction collector (Teledyne ISCO, USA). ACE 3C18-300 4.6х150 mm column (Advanced Chromatography Technologies, UK) was equipped. Chromatography conditions: column flow 1 mL/min, gradient elution from 0% to 30% in 3 min and from 30% to 100% B in 17 min (A: 0.1% (v/v) formic acid in water, B: 0.1% (v/v) formic acid in ACN), column temperature 55°C, injection volume 1000 μL. Fractions were collected with 1AU threshold on 280 nm wavelength.

100 μL of every hydrolyzed sample (*n*=4) were injected. Target peak area was converted into peptide dry mass with molar extinction of Trp, Tyr and Cys under denaturing conditions [18].

### 2.9 HPLC-MS/MS analysis

HPLC-MS/MS analysis was performed on Impact II QqTOF high-resolution mass-spectrometer (Bruker Daltonik, Germany) equipped with Elute UHPLC (Bruker Daltonik, Germany) on Intensity Solo 1.8 C18-2 2.1*100 mm 1.8 µm 90 Å reverse-phase column (Bruker Daltonik, Germany) with following conditions: column flow 0.25 mL/min, gradient elution from 30% to 100% B in 60 min (A: 0.1% (v/v) formic acid in water, B: 0.1% (v/v) formic acid in ACN), column temperature 40°C, injection volume 15 μL (1 µg/µL of dry sample in 60% (v/v) ACN), ESI source in positive mode, HV capillary at 4.5 kV, spray gas – nitrogen at 2.1 bar, dry gas – nitrogen at 8 L/min 220°C, scan range m/z 50-2200, 2 Hz scan rate for full scan, automatic MS/MS mode (CID) with dynamic scan rate 2-8 Hz, nitrogen as collision gas, collision energy from 23 eV at m/z 300 to 65 eV at m/z >1300, automatic internal calibration with ESI-L low concentration tuning mix (Agilent Technologies, USA). Spectra were processed with BioPharma Compass 3.1.1 (Bruker Daltonik, Germany).

### 2.10 Circular dichroism

Collected fractions were dried overnight on rotary vacuum concentrator RVC 2-25 CDplus (Martin Christ, Germany) at 30°C and reconstituted at 2.5 mg/mL concentration in 10 mM sodium phosphate pH 9.3 at 4°C. Solution buffer concentration and pH were adjusted to 30 mM and 6.8 by addition of 1 M pH 6.8 sodium phosphate. The final solution was stored on ice and spectra were collected by Chirascan circular dichroism spectrometer (Applied Photophysics, UK) using 0.1 mm path-length quartz cuvette. Three repetitive scans between 280 and 180 nm were averaged. Accurate peptide concentration was determined as the mean of 215, 210, and 205 nm absorbances, divided by 15, 21, and 32 extinction coefficients respectively and 0.01 cm pathlength. Single spectra analysis and fold recognition was performed using DichroWeb [19] tool. CDSSTR method [20] and dataset 4 [21] were used for secondary structure fraction calculation.

### 2.11 Statistical methods

The linear fit with 95% percentile calculation was performed using OriginPro 2017 (OriginLab Corporation, USA).

SciPy python package was used for mean, standard error and confidence interval calculation, the confidence interval for Student's t distribution was calculated (scipy.stats.t.interval).

## 3 Results

**Fig. 1:**
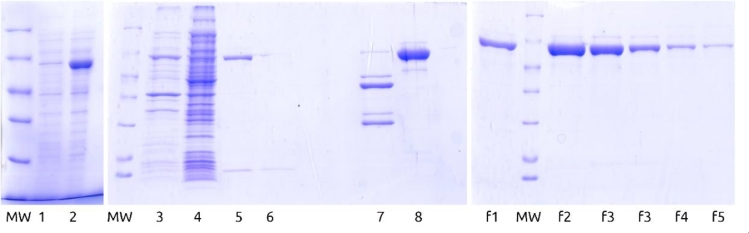
SDS-PAGE of preparative expression, lysis, and purification samples of modified GroEL-enfuvirtide. MW – molecular weight ladder 116, 66.2, 45, 35, 25, 18.4, 14.4 kDa. 1- before and 2 – after expression (25 µL of culture on sample), 3- lysate pellets, 4 – heating pellets, 5 – clarified lysate, 6 - flow through DEAE column 7 – cyanogen bromide hydrolysis products, 8 – purified protein before hydrolysis, f1-f5 – preparative fractions from DEAE column.

*E. coli* BL21(DE3) were transformed with the previously obtained ploop/ES (pET-Duet-1 with modified *T. thermophilus* GroEL-enfuvirtide and intact GroES genes). GroEL-enfuvirtide expression level was 140 (Fig. 1) - 350 mg/L by SDS-PAGE densitometry depending on batch.

Target GroEL-enfuvirtide protein was predominantly soluble after cell lysis and centrifugation. Clarified lysate was heated at 65°C and most denatured host proteins were sedimented by centrifugation. Target protein loss was negligible (Fig. 1, lanes 3–5).

**Fig. A1:**
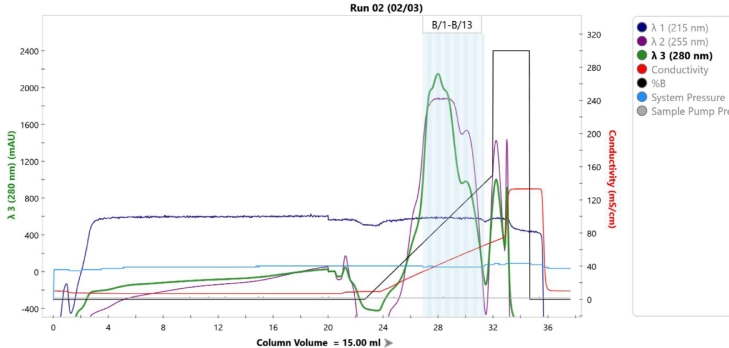
Application and elution chromatogram and fractions example of preparative WAX chromatography.

GroEL-enfuvirtide was readily adsorbed by DEAE resin and eluted at high concentration by sodium chloride gradient (Fig. 1, lanes 6 and f1-f5 and Appendix A, Fig. A1).

**Fig. A2:**
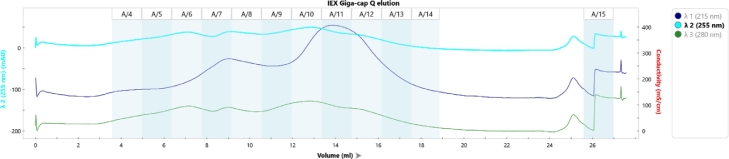
Chromatogram and fractions example of preparative SAX chromatography.

**Fig. A3:**
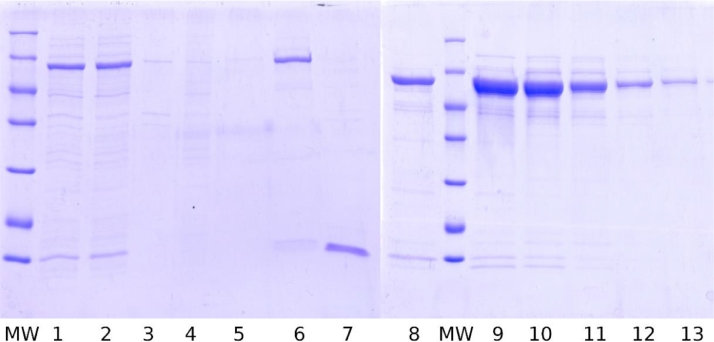
SDS-PAGE gel of lysis and purification samples. MW – molecular weight ladder 116, 66.2, 45, 35, 25, 18.4, 14.4 kDa, 1- harvested cells, 2 – clarified lysate, 3 – lysate pellets, 4 – heating pellets, 5 – column flow through, 6 – column application, 7 – GroES fraction, 8-13 multiple runs summary fractions from 9 to14.

**Fig. A4:**
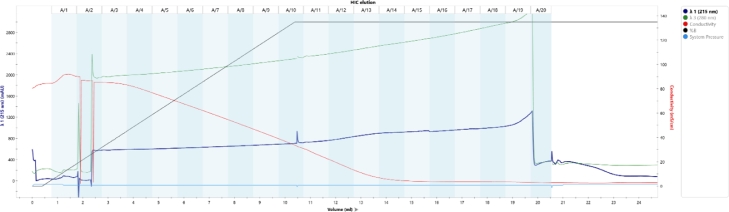
Chromatogram and fractions analytical HIC chromatography on Butyl Toyopearl [41]. 18–20 fractions are 20% 2-propanol addition.

**Fig. A5:**
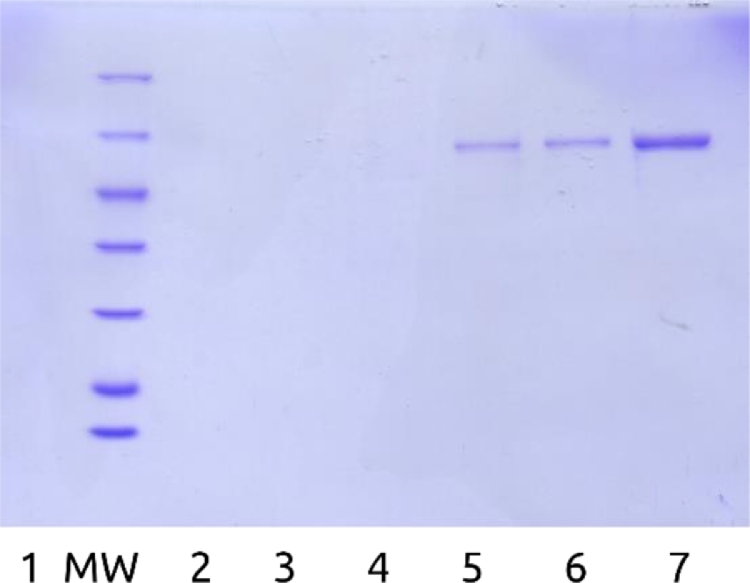
SDS-PAGE gel HIC FT and elution fractions. MW – molecular weight ladder 116, 66.2, 45, 35, 25, 18.4, 14.4 kDa, 1 – FT, 2 – f8, 3 – f10, 4 – f12, 5 – f14, 6 – f16, 7 – f20.

**Fig. A6:**
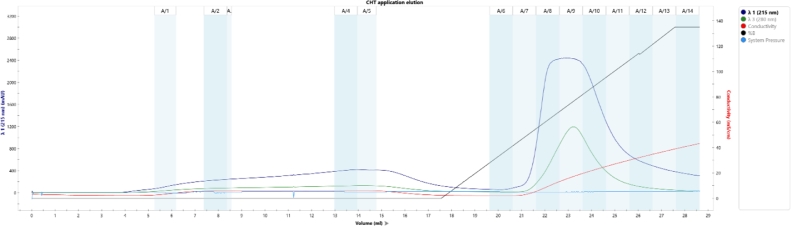
Chromatogram and fractions analytical CHT chromatography on ceramic hydroxyapatite type I. Application in 10 mM 6.8 phosphate buffer and elution with up to 500 mM phosphate gradient.

Alternative chromatography modes were assessed. SAX chromatography was tested under the same conditions. GigaCap Q (Tosoh, Japan) performed similarly with less backpressure but adsorbed GroES additionally (Appendix A, Fig. A2, Fig. A3, ). Ceramic hydroxyapatite type I showed no separation from GroES, and Toyopearl Butyl had excessive retention with substantial sample loss even after 20% isopropanol addition (Appendix A, Fig. A3, Fig. A4, Fig. A5, Fig. A6).

**Fig. 2:**
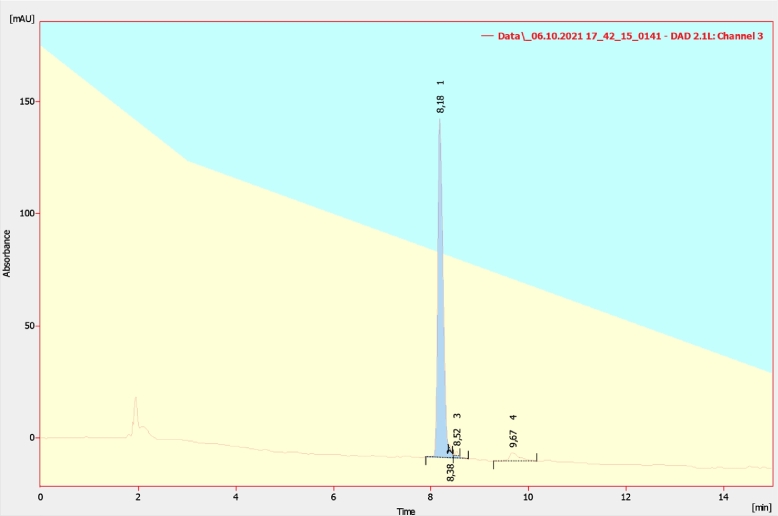
Sample elution profile of purified enfuvirtide on wavelength 280 nm.

**Fig. 3:**
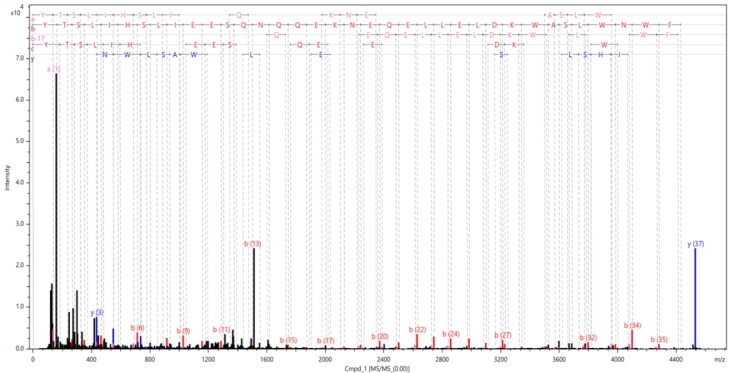
MS/MS spectrum of enfuvirtide Met→Hsl with main fragmentation patterns attributed.

Multiple run fractions were united after SDS-PAGE analysis based on GroEL-enfuvirtide content. United fractions were exchanged into volatile buffer and lyophilized. Lab-scale preparative batch produced 295 mg of 85.4–90% pure target protein from 2.5 liters of culture media. Densitometry data show about 272 (CI 254-290) mg of target protein.

Different cyanogen bromide hydrolysis protocols were tested (Appendix A, Fig. A9). Protein concentration, ultrasound and shaking application, the reaction mixture with or without acetonitrile were tested. Unexpected results were obtained: practically full hydrolysis occurred after 60 min incubation.

The yield of cyanogen bromide hydrolysis and HPLC purification was 33.5–38.9 %, so about 7.14– 8.29 mg of 95% pure peptide (Fig. 2) can be produced from 2.5 L of culture.

HPLC-MS/MS analysis of hydrolyzed mixture confirmed enfuvirtide sequence with additional C-terminal methionine modified by cyanogen bromide cleavage YTSLIHSLIEESQNQQEKNEQELLELDKWASLWNWFM (M37: Met->Hsl) with high probability (Table 1, Fig. 3).

**Table 1:** MS/MS sequence confirmation data for enfuvirtide Met→Hsl.

Measured Mr[Da]	Δ Mr [ppm]	Rel. Int. [%]	RT [min]	Sequence validation	Sequencecoverage
4531.1792	-3.98	100	14.81	97.3%	94.6%

detailed MS2 fragmentation peaks table is presented in Appedix. The circular dichroism spectra of reconstituted peptide were measured (Fig. 4) and secondary structure fraction were calculated (Table 2).

**Table 2:** Enfuvirtide Met→Hsl secondary structure fractions, predicted with DichroWeb server.

Helix1	Helix2	Strand1	Strand2	Turns	Unordered	Total
0.07	0.11	0.16	0.11	0.24	0.32	1.01

## 4 Discussion

Synthetic gene cloning showed that the developed earlier ploop/ES plasmid with BamHI, HindIII and EcoRI polylinker introduced between the codons of amino acids 199 and 201 of modified thermophilic GroEL [16] is a convenient tool for recombinant peptide expression. Also, cultivation parameters for that fusion were generic and there is a great space for protein yield improvement. High density cultures, according to some authors can increase yield up to 9–85 folds [22].

It is known that one of the key parameters for successful and reproducible cell lysis by sonication is energy applied per milliliter of cell suspension. Other crucial sonication parameters, such as cell quantity, buffer content or temperature are much more obvious. In our practice, we used to record the specific presets for one specific sonicator. So, especially for reproducibility purposes and considering initial energy about 500 J/mL [23], [24], [25], a new protocol was successfully tested with little lower energy applied, 300 J/mL. We should mention that energy efficiency of sonicator varies on model, probe, liquid volume and viscosity. In this study, according to device self-measurement, it was about 20% of declared power consumption.

Target protein purification by lysate heating is one of the key advantages of our fusion that retained its thermostability after the introduction of enfuvirtide into thermophilic GroEL sequence. Note that no protease inhibitors were added except EDTA. Addition of other protease inhibitors adds cost to process and can modify target protein during heating [26]. Fast heating of lysate almost immediately after lysis helps skip optimal conditions for proteases and begins denaturing them. The selectivity of heating purification, in our opinion, is comparable with the selectivity of Ni-affinity chromatography in denaturing conditions and cost efficiency is beyond comparison. Our peptide biosynthesis approach seems suitable for peptides with folding issues – it supports a peptide in the solution on the substrate binding surface of GroEL chaperone. Therefore, it is possible to avoid denaturants at all by BrCN cleavage replacement with some cleavage technique in native conditions. However, in terms of biophysics, 65°C heating is above fusion protein melting point [27], so melting reversibility for incorporated peptide should be tested individually.

In previous study we used reverse phase chromatography on C4 resin for fusion protein polishing. Reverse phase in that condition has undeniable advantages: it is universal, has high resolution and yields protein ready for lyophilization though it also has too many drawbacks: protein recovery issues are usually discussed by protein RP phases manufacturers and rarely, by scientists [28]; RP-ready equipment is expensive; it produces organic wastes; it has a moderate binding capacity. Other chromatography modes were tested: strong and weak anion exchange, hydrophobic interactions and hydroxyapatite. The last two were outsiders, they both had moderate binding capacities and no GroEL/GroES separation for hydroxyapatite or recovery issues for hydrophobic interactions resins respectively. DEAE and Q resins have comparable dynamic binding capacity, according to manufacturer – 40 and 47 mg BSA/mL resin, but DEAE did not bind GroES and it was accepted as an advantage. Actual binding capacities for fused protein were not tested. WAX purification protocol was also used for polyphemusin I fusion and for empty fusion partner, modified GroEL. Peak retention varies about 1 CV on gradient, but the method proved to be universal [27].

Target protein desalting by chromatography was chosen. It is a fast and easy procedure; resin is cheap, homemade column can be packed and used under gravity flow. Similarly, dialysis is routine in most biochemical labs and can be used for buffer exchange purposes instead.

The dry weight of purified protein and densitometry data are consistent, which shows low content of non-protein contaminants and buffer residues.

There is a large variety of cyanogen bromide treatment protocols [29], [30], [31], [32]. BrCN hydrolysis needs denaturing conditions to access methionine residues in the protein structure. Every denaturant has its own drawbacks: formic acid both denatures and provides low pH for the reaction, formic acid-based reaction mixture is fully volatile, but it modifies protein with formyl residues; urea and guanidine are nonvolatile, they need hydrochloric acid addition, urea tends to modify protein with carbonylation and guanidine narrows following purification step to RP-HPLC. So, formic acid and minimal reaction time was chosen as reaction conditions. Cyanogen bromide 5 M stock solution in dry acetonitrile helps to minimize exposure to BrCN fumes, additionally this stock is stable and acetonitrile accelerates cleavage [29].

In our opinion, the full and fast cyanogen bromide cleavage is a feature of soluble non-hydrophobic fusion partner [33].

Certain levels of peptide modification in chosen conditions are inevitable, however it is under 5% and can be eliminated during subsequent purification [34].

Large pore analytical-grade C18 sorbent was used for peptide purification. The applicability of flash-RP, SPE-RP, or any other sorbent should be considered for each peptide and each purpose individually. RP HPLC usually uses formic acid (FA) or trifluoroacetic acid (TFA) as mobile phase additives. TFA usually provides better peak shape, but it is potentially toxic [35] and has long environmental lifetime [36]. Formic acid was chosen with full MS compatibility as a bonus.

Total yield for hydrolysis and HPLC step was 36% of theoretical, considering 7.2% share of the free peptide in protein mass and ignoring lyophilized protein purity. Moderate yield can be explained by partial hydrolysis (Fig. 1, lane 7, thin lines above main hydrolysis products) and peptide recovery issues from column and during solubilization.

The cyanogen bromide treatment in 70% formic acid provides fully denaturing conditions, which may cause peptide folding issues. It can be expected that soluble peptide would fold properly on its substrate [37], especially if it is acysteine-free peptide. Enfuvirtide is exceptionally well-studied peptide. The proper fold of enfuvirtide on its substrate is alpha helix [38], but there are circular dichroism data indicating only 18–20% of helical structure for enfuvirtide solutions without substrate [39,40]. We have conducted circular dichroism assessment of enuvirtide solution without substrate. Our data are collected in much more concentrated solution, but total 18% of helical structure is consistent with previously existed data.

Low peptide/fusion protein ratio is a great metabolic burden for host cells. On the other hand, favorable and reproducible fusion protein properties combined with high expression rates – regardless of target peptide – can neutralize that drawback. Substrate-binding domain trimming and peptide tandem repeats can be used to increase peptide/fusion ratio and yield; however, each technique should be tested for each target peptide individually.

Well-characterized, cost-efficient, and straightforward protocol was developed. It has flexibility and most of the methods are scalable up to industrial level. All the basic steps(target peptide sequence cloning, fermentation, host cell denaturing by lysate heating, cyanogen bromide cleavage of fusion protein and reverse-phase peptide purification) are summarized in scheme (Fig. 5). Intermediate purification of fusion protein is optional.

**Fig. 4:**
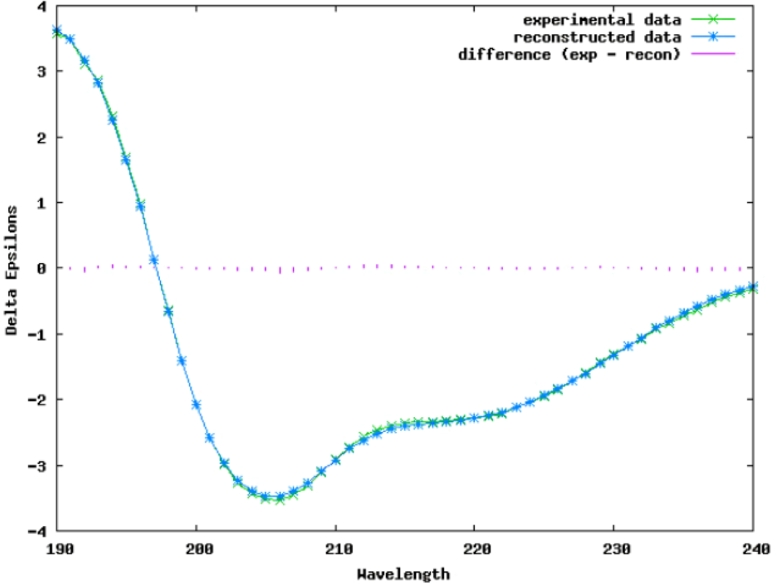
Circular dichroism spectra of enfuvirtide Met→Hsl and theoretical curve, based on predicted secondary structure fractions. The plot was generated by DichroWeb server

**Fig. A7:**
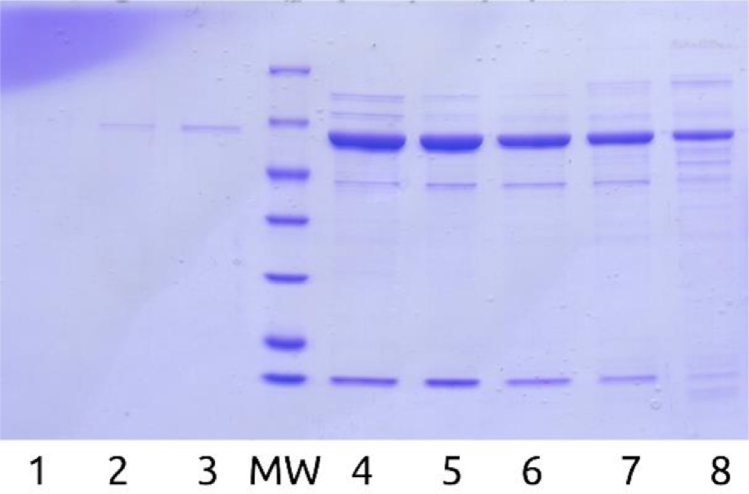
SDS-PAGE gel HCT FT and elution fractions. MW – molecular weight ladder 116, 66.2, 45, 35, 25, 18.4, 14.4 kDa, 1 – f1, 2 – f2, 3 – f4, 4-8 – f7-f11.

**Fig. A8:**
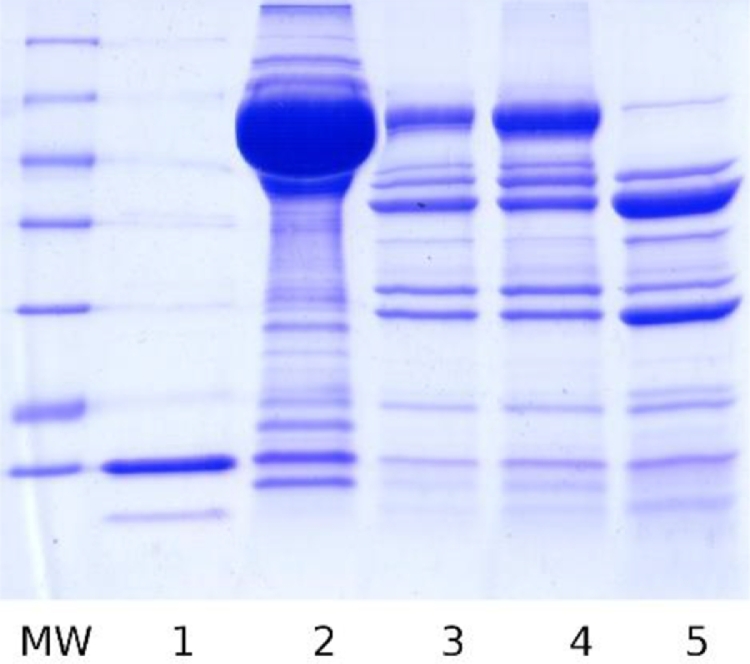
Test cyanogen bromide cleavage results. MW – molecular weight ladder 116, 66.2, 45, 35, 25, 18.4, 14.4 kDa, 1 – GroES after BrCN treatment (5 mg/mL protein, overnight), 2 – modified GroEL-enfuvirtide sample before treatment, 3 – after treatment (25 mg/mL protein, 1h in ultrasound bath), 4 – after treatment (25 mg/mL protein, 2h at vortex), 5 – after treatment (5 mg/mL protein, overnight).

**Fig. A9:**
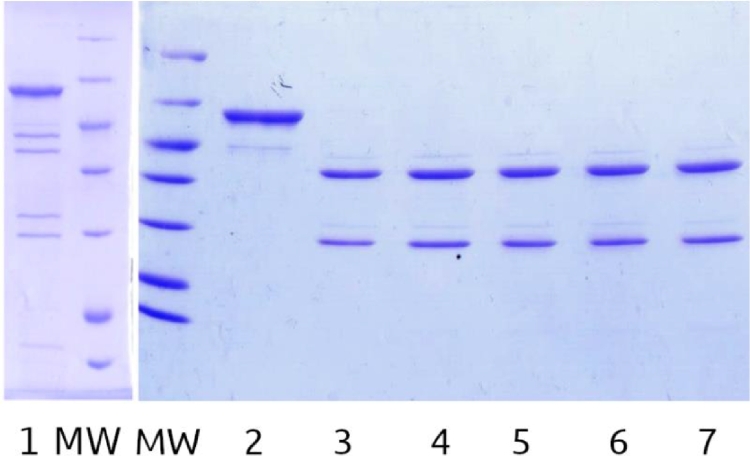
Test cyanogen bromide cleavage results, 5 mg/ml protein, BrCN was added in 5M ACN stock. MW – molecular weight ladder 116, 66.2, 45, 35, 25, 18.4, 14.4 kDa, 1 – modified GroEL-enfuvirtide sample 0h treated, 2 – before treatment, 3-7 – 1-5 h treatment.

The comparison of biophysical properties of the fusions of modified GroEL with different peptides [22] and full quality and biological activity assessment of synthesized enfuvirtide [29] are the prior directions for future study. Also, systematic practical comparison with popular fusion tags is unavoidable to name our project “the new fusion system” (Fig. A1, Fig. A2, Fig. A3, Fig. A4, Fig. A5, Fig. A6, Fig. A7, Fig. A8, Fig. A9, Table A1).

**Fig. 5:**
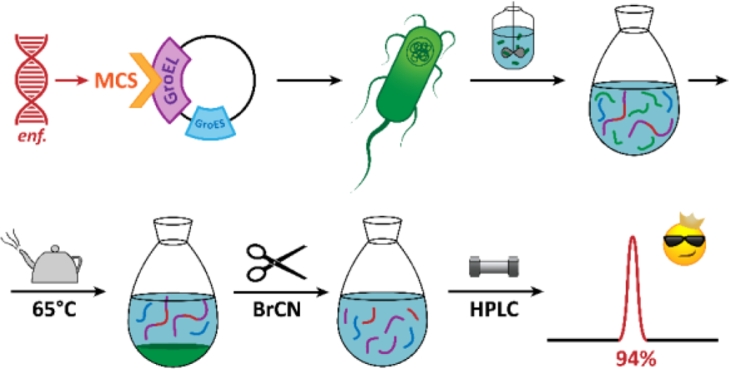
Basic peptide biosynthesis scheme.

**Table A1:** MS2 fragmentation peaks of enfuvirtide.

## Funding

The reported study was funded by RFBR, project number 18-29-08023.

## Data availability statement

All stock solutions protocols, any other mentioned protocols and data are available in details on request from corresponding author

## Author contributions

**Vladimir Zenin**: conceptualization, methodology, data curation, formal analysis, writing—original draft preparation, **Maria Yurkova**: conceptualization, methodology, writing—review and editing, supervision **Andrey Tsedilin**: methodology, writing—original draft preparation, writing—review and editing, **Alexey Fedorov**: conceptualization, writing—review and editing, funding acquisition, supervision

## Declaration of Competing Interest

The authors of manuscript “Enfuvirtide biosynthesis in thermostable chaperone-based fusion” Vladimir Zenin, Maria Yurkova, Andrey Tsedilin and Alexey Fedorov declare no conflict of interest.
